# Chronic Obstructive Lung Disease: Treatment Guidelines and Recommendations for Referral and Multidisciplinary Continuity of Care

**DOI:** 10.3390/jcm13020303

**Published:** 2024-01-05

**Authors:** Javier De Miguel-Díez, Alberto Fernández-Villar, Esperanza Doña Díaz, Marta Padilla Bernáldez, Eva Trillo-Calvo, Jesús Molina París, Miriam Barrecheguren, José Miguel Valero Pérez, María Teresa Ramírez Prieto

**Affiliations:** 1 Servicio de Neumología, Hospital General Universitario Gregorio Marañón, Instituto de Investigación Sanitaria Gregorio Marañón (IiSGM), 28007 Madrid, Spain; 2 Departamento de Medicina, Facultad de Medicina, Universidad Complutense de Madrid, 28040 Madrid, Spain; 3 Servicio de Neumología, Hospital Álvaro Cunqueiro, Grupo NeumoVigo, Instituto de Investigación Sanitaria Galicia Sur, Centro de Investigación Biomédica en Red de Enfermedades Respiratorias (CIBERES), 36312 Vigo, Spain; jose.alberto.fernandez.villar@sergas.es; 4 Unidad de Asistencia al Paciente EPOC, UGC Médico-Quirúrgica de Enfermedades Respiratorias, Hospital Regional Universitario de Málaga, 29010 Málaga, Spain; esperuli@gmail.com; 5 Hospital Universitario 12 de Octubre, 28041 Madrid, Spain; mpadillab@salud.madrid.org; 6 Institute for Health Research Aragón, Centro de Salud Campo de Belchite, 50130 Belchite, Spain; evatrillo83@hotmail.com; 7 Centro de Salud Francia, Dirección Asistencial Oeste, 28943 Fuenlabrada, Spain; jmolinaparis@gmail.com; 8 Servicio de Neumología, Hospital Universitari Vall d’Hebron, 08035 Barcelona, Spain; miriam.barrecheguren@vallhebron.cat; 9 Centro de Salud Bétera, 46117 Bétera, Spain; jmvalerp@gmail.com; 10 Servicio de Neumología, Hospital Universitario Infanta Sofía, 28702 Madrid, Spain; mtramirezp@salud.madrid.org

**Keywords:** chronic obstructive pulmonary disease, continuity of patient care, referral and consultation

## Abstract

Chronic Obstructive Pulmonary Disease (COPD) constitutes a major public health problem, and it is anticipated that its prevalence will continue to increase in the future. Its progressive nature requires a continuous and well-coordinated care approach. The follow-up for COPD should primarily focus on disease screening and control, which includes monitoring of pulmonary function, prevention of exacerbations, identification of aggravating factors and comorbidities, as well as ensuring treatment adequacy and adherence. However, existing clinical practice guidelines and consensus documents offer limited recommendations for the follow-up. In this context, we undertake a review of COPD treatment and the continuity of care recommendations endorsed by several scientific societies. Moreover, we underscore the importance of the involvement of nursing and community pharmacy in this process, as well as the utilization of quality indicators in the provision of care for the disease.

## 1. Introduction

Chronic obstructive pulmonary disease (COPD) is a chronic, often progressive, lung disorder causing persistent airflow obstruction. It is characterized by respiratory symptoms such as dyspnoea, cough, and sputum production associated with airway and/or alveoli alterations. The aetiology of COPD is primarily linked to long-term exposure to noxious particles and gases, with tobacco smoke being the most common risk factor. Nevertheless, environmental factors, such as air pollution, occupational exposure, and genetic predisposition, also contribute to its development [1]. Despite being both preventable and treatable [1], COPD poses a substantial health, economic, and social burden, emerging as a major public health problem [1,2]. Patients afflicted with this pathology experience a reduced quality of life due to chronic respiratory symptoms, physical limitations, and psychological distress [3] that can lead to disability, early retirement, productivity loss, healthcare costs, and caregiver burden [4]. In 2019, it accounted for 212.3 million prevalent cases, 3.3 million deaths worldwide, and 74.4 million disability-adjusted life years (DALYs), making it the third most common contributor to global mortality [2]. According to extensive epidemiological studies, the global prevalence of COPD among people aged between 30 and 79 is approximately 10.3% (95% confidence interval [CI] 8.2–12.8%) [1]. In Spain, COPD continues to be very prevalent, with values of 11.8% in people over 40 years of age. Likewise, underdiagnosis rates are elevated (74.7%) higher in women than in men (80.6% vs. 70.4%, *p <* 0.001) [5]. 

It is believed that part of this under-recognition is due to the nonspecific nature of COPD symptoms, which may lead healthcare providers to not recognize COPD patients with symptoms different from the classic ones [6].

Some guiding points to establish suspicion of COPD include [6]: age > 35 years, history of smoking ≥ 10 pack years, exposure to risk factors: smoking, environmental, or host factors, dyspnoea, and chronic cough, with or without sputum.

If COPD is suspected, patients should undergo a clinical assessment based on pulmonary function studies and other treatable conditions [6].

Given the rising rates of smoking in low- and middle-income countries (LMICs) [1] among women and younger patients, as well as new vaping and heating devices for tobacco [7], and the aging populations in high-income countries, it is anticipated that the prevalence of COPD will continue to increase in the future [1].

The progressive nature of chronic illnesses requires a continuous and coordinated care approach. Continuity of care is a crucial element of support for patient-centred quality care. Collaboration between healthcare allows for optimal symptom management, reduces the number of disease exacerbations, and decreases hospital visits [8]. 

Recently, SEMERGEN et al. have presented an updated document on COPD referral criteria and continuity of care drawn up by several scientific societies [6]. 

## 2. Treatment of Stable COPD

The basis of pharmacological treatment are inhaled drugs [9]. These include long-acting bronchodilators (LAB; beta2-agonists [LABA] or long-acting anticholinergics [LAMA]), short-acting bronchodilators (SAB; beta2-agonists [SABA] and short-acting anticholinergics [SAMA]), and inhaled corticosteroids (IC). In addition to bronchodilator therapy, other treatments such as theophylline, roflumilast, and mucolytics can be used [9]. 

Every pharmacological treatment plan must be personalized. Factors that should be considered are the severity of symptoms, risk of exacerbations, adverse effects, concurrent medical conditions, accessibility and affordability of medications, as well as the patient’s response, inclinations, and ability to utilize different drug administration devices [1]. 

A distinction can be made between initial treatment when the patient has just been diagnosed with COPD and follow-up treatment when the patient was previously treated but does not obtain appropriate response [10,11]. In the following, recommendations for the treatment of stable chronic obstructive pulmonary disease (COPD) will be presented. It should be emphasized, however, that some ideal characteristics will also be mentioned for those with a recent COPD exacerbation.

### 2.1. Initial Treatment

**Figure 1:**
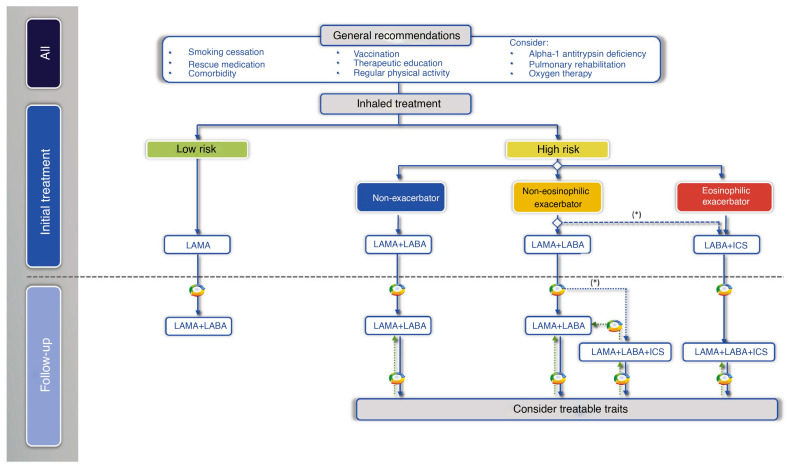
Treatment of COPD guided by risk level and phenotype. (*) Second-line in patients with blood eosinophils > 100 cells/mm^3^, according to the frequency, severity, and aetiology of the exacerbations, assessing the risk of pneumonia. Reproduced from GesEPOC guidelines [9].

Regarding initial pharmacological treatment, the Spanish COPD guidelines (GesEPOC) propose an inhaled treatment guided by symptoms or clinical phenotype according to the patient’s risk. “Low risk” are those with FEV1 ≥ 50%, dyspnoea (mMRC) of 0 or 1 and one or no exacerbation in the previous year (without hospitalisation). If any of these requirements are exceeded, they will be classified as “high risk”. In the low-risk patient with permanent respiratory symptoms or those that limit daily tasks, the basis of pharmacological treatment should be LAB, preferably LAMA over LABA. On the other hand, patients at high risk should be differentiated by phenotype: in the case of a non-acute and non-eosinophilic patient, treatment should be initiated with LABA + LAMA, and in the case of an eosinophilic patient, treatment should be initiated with IC + LABA [9] (Figure 1).

**Figure 2:**
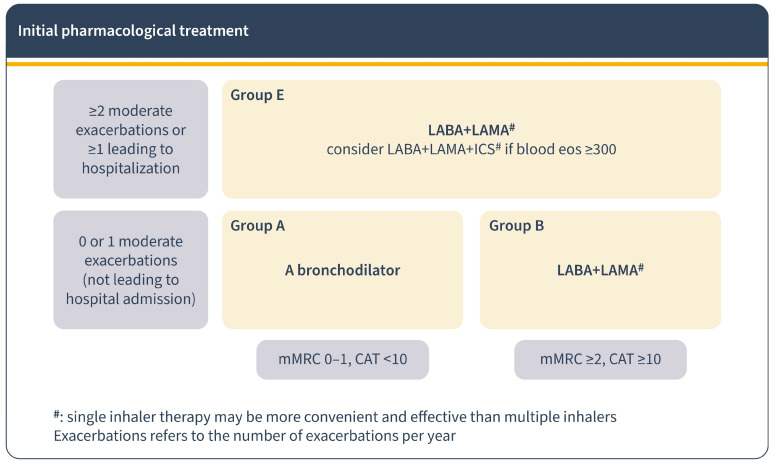
Pharmacological treatment for COPD regarding GOLD report 2023. Initial pharmacological treatment.

According to the 2023 GOLD report, initial treatment is based on future risk, based on symptomatology and exacerbations. In patients with permanent symptoms, it recommends starting directly with dual LAB. If IC is necessary, it is recommended to combine it with LABA + LAMA rather than with LABA alone. Also, it proposes to initiate triple therapy (LABA + LAMA + IC) in patients with ≥300/µL blood eosinophils consistently and two or more moderate exacerbations or in those whose onset required an admission [1] (Figure 2).

### 2.2. Follow-Up Treatment

**Figure 3:**
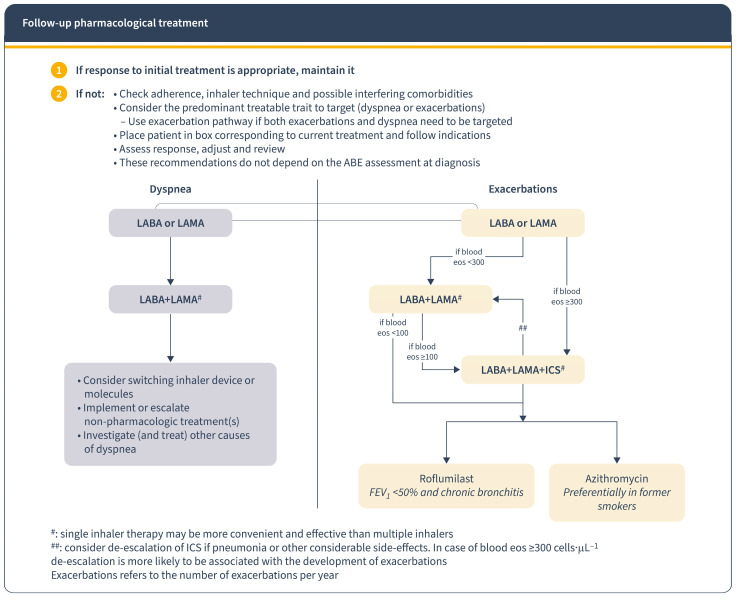
Follow-up pharmacological treatment regarding GOLD report 2023. Reproduced from GOLD guidelines [1]. Eos: blood eosinophil count in cells per microliter; mMRC: modified Medical Research Council dyspnoea questionnaire; CAT™: COPD Assessment Test™.

In the management of COPD patients, the response to initial treatment determines subsequent approaches. If the response is positive, treatment continues; if not, the focus shifts to controlling the predominant symptom [12]. GesEPOC guidelines recommend personalized treatment based on risk factors and symptoms. For low-risk patients with inadequate control, dual bronchodilation therapy is advised. High-risk patients on dual bronchodilation may need triple therapy (LABA + LAMA + CI) in a single inhaler [9] (Figure 1). The GOLD 2023 report emphasizes adjusting treatment according to dyspnoea and exacerbation presence and using blood eosinophil count as a biomarker for CI use against exacerbations [1] (Figure 3). Regular assessment of symptoms and exacerbations and patient assessment is crucial to ensure stability and adjust treatment if needed [12].

### 2.3. Treatable Traits

There are other aspects to be assessed beyond pharmacological treatment for a comprehensive approach to COPD [13,14]. Patients with COPD may also exhibit additional nonspecific symptoms and signs. These are treatable aspects that require attention, particularly in individuals at high risk [9]. 

Encouraging educational and self-management strategies that motivate, engage, and educate patients to improve their health behaviours has a positive impact on health-related quality of life, exacerbation duration, hospitalizations, and healthcare utilization [14,15]. Non-pharmacological therapy has been shown to improve the status and/or prognosis of COPD patients in relation to smoking cessation, influenza and pneumococcal vaccination, improved nutrition and physical activity, as well as in advanced COPD, pulmonary rehabilitation, long-term oxygen therapy, non-invasive mechanical ventilation, or surgical techniques (lung transplantation, volume reduction surgery, bullectomy) [13,16,17]. 

## 3. Continuity of Care in EPOC Patients

**Figure 4:**
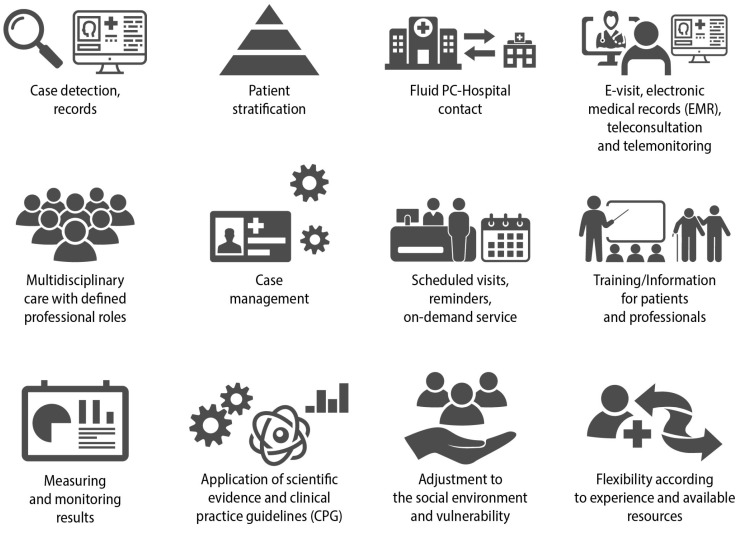
Key elements for adequate continuity of care in COPD. Reproduced from SEMERGEN, SEPAR, semFYC, SEMG, SEFAC, GRAP. Referral criteria in COPD. Continuity of care. IMC 2023 [6].

The concept of continuity of care, particularly in primary care (PC) settings, involves delivering consistent and patient-centred support to an individual over an extended period, addressing both health and illness needs. When dealing with the management of chronic conditions, it can be perceived as the logical and timely delivery of services by different providers [8]. COPD requires multidisciplinary health care where the different levels of care cooperate in a coherent and well-coordinated manner (Figure 4) [18,19]. 

The PC professional is the predominant provider of care for patients with COPD, as most patients do not have specific needs. However, due to the heterogeneity of their presentation, bidirectional communication with specialized care, mainly with pulmonologists, is necessary [20,21]. Thus, general practitioners must be well trained to recognize when to refer to other levels of care [21]. 

**Figure 5:**
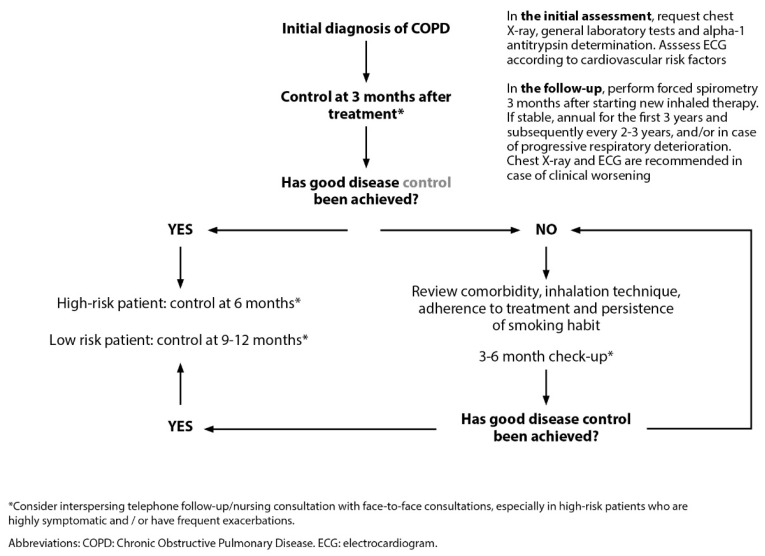
Follow-up of stable COPD patients. Reproduced from SEMERGEN, SEPAR, semFYC, SEMG, SEFAC, GRAP. Referral criteria in COPD. Continuity of care. IMC 2023 [6].

Continuity of care would allow for early detection of exacerbations, aggravating factors or co-morbidities, correct treatment errors, and enhanced self-care, reducing hospitalization rates and associated costs [19]. Unfortunately, clinical practice guidelines and consensus documents offer insufficient recommendations for the follow-up of these patients [1,9,22,23,24]. The document on COPD referral criteria and continuity of care drawn up by several scientific societies offers guidelines on the periodicity of COPD follow-up in coordination between PC and hospital care (Figure 5) [6]. The first follow-up should take place 3 months after the initial diagnosis of COPD and implementation of the necessary treatment. In cases where optimal control has not been achieved, possible causes should be assessed and medication should be adjusted. A new assessment should be performed in the following 3-6 months. In cases with good disease control, follow-up will be conducted at 6 months in high-risk patients and at 9–12 months in the case of low-risk profiles. The time periods given will be adapted according to the clinical situation of the patient, shortening the follow-up interval in the most severe or uncontrolled subjects. After hospital discharge due to exacerbation, close follow-up should be carried out (48–72 h) [6]. 

SEMERGEN et al. recommend performing forced spirometry annually during the first three years after diagnosis with the aim of detecting individuals with accelerated loss of function. Afterward, this should be performed every 2–3 years. In case of progressive clinical deterioration with changes in treatment, it should be performed within 3 months since it can provide information about the cause of clinical worsening as well as the impact of the therapy modification, respectively. Furthermore, exacerbations of COPD (COPD exacerbation syndrome, CES) can occur, marked by a deterioration in respiratory symptoms. These patients should undergo revaluation within 48–72 h, and in severe cases, follow-up assessments should be conducted at 2–4 weeks and 8–12 weeks [6]. 

High-quality spirometric measurements are achievable in any healthcare environment, and it is crucial that all healthcare professionals involved in COPD patient care have access to them [1]. Ancochea et al. [19] already proposed in 2021 a spirometry training plan focused not only on primary care but also on nursing and community pharmacies. Its aim would be to encourage early diagnosis to try to reduce the under-diagnosis of COPD. 

Several aspects should be checked during follow-up visits (Table 1). Symptoms and exacerbations should be assessed by the Clinical Control Questionnaire [9]. In particular, dyspnoea degree must be determined using the Modified Medical Research Council (mMRC) scale [25] and its causes by diagnostic tests (chest computed tomography, echocardiogram) [6]. Another questionnaire is the CAT test, which enables the measurement of patients’ quality of life. Variations ≥ 10 points require evaluation by a physician. Referral is also essential when there has been a loss of greater than 10% of weight within the preceding six months or when the individual’s body mass index (BMI) falls below 21 kg/m^2^ [6].

Forced spirometry is used to measure lung function. It serves to adjust patient’s treatment and acts as a prognostic factor. FEV_1_/FEV_6_ values *<* 0.75 indicate possible obstruction, and the patient should be referred to the doctor [6]. If needed, oxygen therapy should be adjusted to maintain a PaO_2_ ≥ 60 mmHg or oxygen saturation ≥ 90% at rest and at sea level. It should be maintained for >15 h per day [9]. 

Also, inhaler review should be performed every 2 months [6]. It is recommended to minimize the number of doses and devices as much as possible. In each visit, it is necessary to check the patient’s satisfaction with the inhaler and ensure that the technique used is appropriate [1,14,26,27]. The in-check dial G16^®^ can be used to check if the patient has an adequate inspiratory flow to use the inhalation device [6]. Psychological and socioeconomic problems should be determined as they can condition therapeutic control [1,6,28]. 

Additionally, vaccination calendar must be updated since respiratory infections are an aggravating factor in COPD. Influenza and SARS-CoV-2 are assessed according to campaign or recommendations [1,9]. Concerning the pneumococcal vaccine for individuals with COPD, it is advisable to prioritize the 20-serotype conjugate vaccine (PCV20). In cases where the sequential vaccination schedule is unavailable, the conjugated vaccine VCN15 should be administered first, followed by PPSV23 polysaccharide vaccine within a minimum of 8 weeks and a maximum of 1 year. Those who have previously received the sequential schedule would be considered correctly vaccinated, without the need for PCV20 [6]. Tetanus, diphtheria, and pertussis (Tdap) vaccine should be updated in those not vaccinated during adolescence [1] and Herpes zoster vaccine in patients over 50 years [6].

Telemedicine (TM) represents a new option for these follow-up visits. It appears to be a promising approach to patient care in chronic illnesses [29]. However, recent research has produced mixed findings regarding its effectiveness for COPD patients. Some studies suggest potential benefits [30,31,32], while others indicate that TM is unlikely to lead to statistically significant improvements [33,34]. TM interventions have not shown any evidence of harm. Therefore, they could be a valuable additional healthcare resource, tailored to individual needs [34].

This continuous monitoring process involves not only general practitioners and pulmonologists but also other people such as nurses, physiotherapists, rehabilitators, emergency physicians, caregivers, and others… [21].

## 4. Role of Nursing in the Management of Patients with COPD

The role of the nurse is essential in the management of the COPD patient’s condition, both in the assessment and monitoring of COPD, as well as in providing guidance, health education, and support to people with the disease and their families [35,36]. Their intervention in disease management has demonstrated improvements in quality of life, emotional state, hospital admissions, and physical capacity of affected patients [36]. 

A key figure to be consolidated is the continuity of care nurse. The nurse ensures continuity of care for patients throughout their care pathway. Their functions should include the management of incidents related to continuity of care and care continuity, delays in care, performance of tests, management of appointments, coordination between professionals, etc. [37]. 

### 4.1. Initial Assessment

In the initial assessment it is necessary to know the level of knowledge [28], perception, and control of the disease and to assess the treatment adherence, so as to detect inadequate behaviours in the management [28].

It is important to collect information on previous and current risk factors (smoking, alcohol, changes in mood [28,38], exposure to gases or other irritants [6]), unhealthy habits that affect the progression of the pathology [38], and number of admissions and exacerbations in the last year [28]. In addition, prescribed medication, vaccination, vital signs (oxygen saturation, respiratory rate), comorbidities, and patient symptoms (mMRC scale, CAT test, COPD clinical questionnaire) should be recorded [6,28]. The patient’s autonomy and social environment should also be assessed. 

### 4.2. Management and Follow-Up

Based on the results obtained, a plan for patient follow-up and education will be drawn up, in which individual needs should be prioritized [6]. 

The educational plan should include smoking cessation, training in inhaled therapy, and recommendation of vaccination and healthy lifestyles. It should promote the prevention of complications and exacerbations by explaining how to protect oneself, recognize them, and take action [36]. 

It is essential to establish follow-up visits to review the aforementioned points. In addition, these visits should include the patient in programs to improve physical condition and exercise tolerance, nutritional status, and even social readaptation services, if necessary [38]. 

Exercise programs for the development of cardiopulmonary fitness, muscle strength, and flexibility have been recommended by the American College of Sports Medicine, the Thoracic Society, and the European Thoracic Society [39]. Exercise therapy is supported by a greater level of evidence in pulmonary rehabilitation as potentially reducing dyspnoea symptoms, enhancing motor skills, improving psychiatric problems, and improving quality of life [40]. Physical activity is not recommended during an exacerbation or in extreme environmental situations [41]. 

Regarding nutritional status, both obesity (BMI > 30 kg/m^2^) and malnutrition (BMI *<* 18.5 kg/m^2^) have a negative impact on the disease. Dietary advice based on the Mediterranean diet is recognized by the international scientific community [42]. Vitamin D supplementation in these patients has been shown to have benefits on exacerbations where levels were *<* 25 mmol/L [13]. In addition to dietary advice and nutrition supplementation (as higher protein intake) screening for malnutrition should also be routinely performed [43]

## 5. Role of the Community Pharmacy in the EPOC Patients’ Continuity of Care

The community pharmacy is easily accessible and perceived to be affordable by the public, making it often the first point of contact in the healthcare system [44,45]. The role of the pharmacist has evolved beyond mere medication adjustments and now encompasses a diverse range of patient-centred health management tasks [45]. 

They play a crucial and emerging role in managing COPD by providing a range of services and support to affected patients [46,47]. These include prevention, screening potential patients, disease counselling, comprehensive disease management, and education [45].

### 5.1. Prevention 

The community pharmacists can participate in primary prevention by offering pharmaceutical advice on smoking cessation, vaccination recommendations, and the importance of following healthy lifestyle habits [46,47]. 

### 5.2. Screening

They also play a key role in the screening and early detection of patients with COPD, detecting symptoms compatible with the disease and referring patients to physicians for proper diagnosis and treatment prescription [46]. 

In the pharmacy, it is recommended to use the “Chronic Obstructive Pulmonary Disease-Population Screener” test (COPDPS) [48] in users over 35 years of age who are smokers or ex-smokers with chronic respiratory symptoms. This questionnaire consists of 5 questions on respiratory function and the ability to perform some daily activities, and each question is scored between 0 and 2. With values ≥ 4, referral to the PC team for spirometry is recommended. 

**Figure 6:**
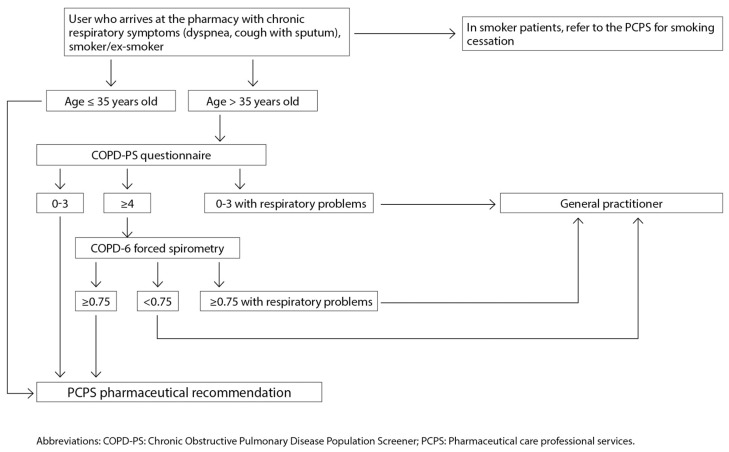
COPD screening algorithm in community pharmacy. Reproduced from SEMERGEN, SEPAR, semFYC, SEMG, SEFAC, GRAP. Referral criteria in COPD. Continuity of care. IMC 2023 [6]. PPAS, Professional Pharmaceutical Assistance Service.

However, validated portable pulmonary function meters or more sophisticated spirometers can be used beforehand to make the referral to the family physician more specific. Forced spirometry with a validated pulmonary function device showing FEV_1_/FEV_6_ values *<* 0.75 indicates a possible obstruction, and a physician referral should be made (Figure 6). If the ratio is greater than or equal to 0.75 in smoking patients, smoking cessation is recommended, explaining the benefits associated with their specific situation and referring them internally to the smoking cessation service. 

In addition, in the case of values that do not indicate obstruction, if the user presents respiratory symptoms, a referral should be made for evaluation by the family physician. In these cases, if after some time, the user continues to present criteria of possible COPD, the screening tests for the disease should be performed again [6].

### 5.3. Education, Follow-Up, and Referral

Community pharmacies serve as the primary point for patients to obtain their prescribed medications. Pharmacists ensure that there are not medication discrepancies and that patients understand how to take their medications [47]. 

They can provide educational information to patients about COPD, its causes, and triggers, as well as guidance on how to use inhalers. They teach patients proper inhaler techniques and review them at least once every 2 months [27,49,50], which are crucial to ensure treatment efficacy. If after detection of misuse of the inhalation device and training, the patient fails to use the device properly, the patient should be referred to the PC team to evaluate a possible change [6]. 

Also, they can track symptoms and treatment adherence (TAI questionnaire and electronic prescription medication withdrawal record), which can be valuable for adjusting treatment plans and preventing exacerbations. In case of exacerbations or moderate to severe adverse reactions, the family physician should be consulted [6]. 

They should work collaboratively with other healthcare professionals, such as physicians and nurses, to ensure comprehensive and coordinated care for COPD patients. 

## 6. Importance of Quality Indicators in the Management of COPD Care

The field of quality measurement in healthcare has become increasingly prominent, drawing interest from a wide range of stakeholders, including researchers, policymakers, and the general public. This heightened attention is driven by a shared objective: to establish more structured and comprehensive approaches to assess and compare the quality of care offered by diverse healthcare providers. 

Researchers and policymakers are at the forefront of these efforts, working collaboratively to refine the methods and tools used for quality measurement. The goal is to create a standardized framework that allows for the objective evaluation of healthcare quality across different institutions and settings. This standardization aims to facilitate fair comparisons and identify areas where improvements are needed [51].

Healthcare quality is a fundamental aspect of how well a healthcare system performs. It involves providing effective, safe, and patient-centred care [52]. Quality indicators (QI) are widely used to improve the quality of health care. The criteria for their selection should be the importance of the subject and that they are evidence-based. It is essential that it is measurable from available high-quality data, easy to calculate and interpret [53]. 

High quality care for COPD patients is important, mainly because of the severe consequences and increasing prevalence of COPD [54,55]. Clinical practice guidelines (CPGs) have helped to improve the quality of care, although their current compliance is still insufficient, with strong variations being detected among professionals [54]. The following indicators are proposed for COPD assessment. These were extracted from the guide created by experts from the main Spanish scientific societies, which encompass the professionals who deal with these patients [6] (Table 2).

## 7. Conclusions

Continuity of care is a fundamental approach to prevent readmissions and potentially enhance health outcomes for COPD patients. In COPD, clinical practice guidelines and consensus documents offer insufficient recommendations for the follow-up. Here, we review COPD referral criteria and continuity of care recommendations by several scientific societies. Continuity of care in COPD settings involves different healthcare professionals (including nurses and community pharmacists) and levels of care. Follow-up of stable COPD should focus on disease screening and control (monitoring of pulmonary function, prevention of exacerbations, identification of aggravating factors and comorbidities) and treatment adequacy and adherence. The basic pharmacological treatment in COPD is inhalation devices. Improving compliance is related to simplifying the number of doses and devices, assessing patient satisfaction in the choice of devices, and checking the inhalation technique (every two months) and degree of adherence at each visit. In addition, intervention for smoking cessation, adoption of healthy lifestyles, and updating of vaccinations is essential. Telemedicine can be a useful tool in the follow-up of COPD patients, especially in those with stable COPD. Likewise, the patient should participate in this process, so it is interesting to offer personalized health education about the disease and treatment, how to manage it, how to recognize problems, and how to deal with them.

## Figures and Tables

**Table 1:** Follow-up activities in patients with COPD coordinated between the different care settings. Adapted from SEMERGEN, SEPAR, semFYC, SEMG, SEFAC, GRAP. Referral criteria in COPD. Continuity of care. IMC 2023 [6].

Clinical Evaluation	
Disease control (symptoms and exacerbations)	Clinical Control Questionnaire for COPD - Patient’s status compared to the previous visit - Exacerbations in the last 3 months - Excessive use of rescue medication - Evaluation of sputum (Murray Scale) - Exercise in the last week - Dyspnoea degree and cause (mMRC Scale)
Patient’s quality of life	CAT Test
Assessment of body mass index (BMI)	Both obesity (BMI > 30 kg/m^2^) and malnutrition (BMI *<* 18.5 kg/m^2^) are associated with a worse prognosis in COPD.
Monitoring the appearance or presence of what it calls “treatable features” associated with COPD	Alpha-1 antitrypsin deficiency, dyspnea, chronic bronchitis, severe emphysema and pulmonary hyperinflation, chronic bronchial infection, bronchiectasis, precapillary pulmonary hypertension, chronic respiratory insufficiency, cachexia.
Complementary tests	
Lung function	Forced spirometry
Determination of blood pressure, heart rate, and oxygen saturation	In the case of chronic respiratory failure (arterial blood gas: PaO_2_ *<* 60 mmHg and/or PaCO_2_ > 45 mmHg), consider continuous home oxygen therapy or non-invasive ventilation.
Treatment	
Treatment adherence and evaluation (response and adverse effects)	Follow-up with the TAI questionnaire and record of medication withdrawn from the electronic prescription.
Inhaler technique review	Every 2 months to check patient’s satisfaction and ensure appropriate.
Review of vaccination status	Influenza, SARS-CoV-2, pneumococcal, herpes zoster and tetanus, diphtheria, and pertussis (Tdap).
Health education	
Nutritional status	
Recommendation for regular physical activity adapted to the clinical situation	Calculation of the level of physical activity using the brief CGPPAQ-EGPPAQ2 questionnaire (Spanish version of the Brief Physical Activity Assessment Tool) and assessment of sedentary time (hours/day).
Persistence of active smoking	Address smoking cessation, and if not achieved, refer to the Smoking Cessation Service.

BMI, body mass index; CAT, COPD assessment test; COPD, chronic obstructive pulmonary disease; mMRC, modified Medical Research Council; PaCO_2_, partial pressure of arterial carbon dioxide; PaO_2_, partial pressure of arterial oxygen; TAI, test of adherence to inhalers.

**Table 2:** Quality indicators in COPD. Created from SEMERGEN, SEPAR, semFYC, SEMG, SEFAC, GRAP. Referral criteria in COPD. Continuity of care. IMC 2023 [6].

Indicator Name	Formula	Objective	Exclusions
**Indicators for COPD initial diagnosis**			
Percentage of patients with COPD correctly diagnosed	(Number of COPD patients diagnosed with spirometry with BD test/Total number of patients diagnosed with COPD) × 100	>85%	Patients unable to perform maneuvers
Percentage of patients with suspected and/or confirmed COPD with PA and LAT chest X-ray at the beginning of the study	(Number of patients with PA and LAT chest X-ray performed at the time of COPD assessment/Total number of patients diagnosed with COPD) × 100	>95%	Patients refusing to undergo imaging test
Percentage of COPD patients with AAT level determination	(Number of COPD patients diagnosed and tested for AAT/Number of patients diagnosed with COPD) × 100	>95%	Patients refusing to undergo the test
**Indicators for COPD treatment and control**			
Percentage of COPD patients offered smoking cessation assistance	(Number of COPD patients who are active smokers and documented to be offered smoking cessation assistance/Total number of active smoking COPD patients) × 100	>85%	None
Percentage of COPD patients prescribed vaccinations according to the current vaccination schedule	(Number of COPD patients with a prescription for vaccination according to the current vaccination schedule/Total number of COPD patients) × 100	>85%	Patient refusal of vaccination
Percentage of COPD patients with a regular exercise program	(Number of COPD patients advised to follow a regular exercise program/Total number of COPD patients) × 100	>85%	Patient refusal or inability to participate
Percentage of COPD patients with inhaler technique review	(Number of COPD patients whose inhaler technique is reviewed/Total number of COPD patients) × 100	>85%	Inability to perform the review and absence of a caregiver
Percentage of COPD patients with inhaler adherence review	(Number of COPD patients whose inhaler adherence is reviewed/Total number of COPD patients) × 100	>85%	Inability to perform the review and absence of a caregiver
Percentage of COPD exacerbators with microbiological control	(Number of COPD exacerbators with sputum culture for bacteria and mycobacteria/Total number of COPD exacerbators) × 100	>85%	Inability to collect samples
**Indicators for Continuity of Care of COPD Patients**			
Percentage of COPD patients with home oxygen with scheduled reviews in Pulmonology	(Number of COPD patients with home oxygen with scheduled reviews in Pulmonology at least every 6 months/Total number of COPD patients with chronic home oxygen) × 100	>90%	Significant mobility limitations
Percentage of COPD patients with functional deterioration referred to Pulmonology from Primary Care	(Number of COPD patients with functional deterioration in the previous three months referred to Pulmonology from PC/Total number of COPD patients with functional deterioration in the previous three months) × 100	>90%	Patient refusal of referral
Percentage of COPD patients with radiological alert referred to Pulmonology from Primary Care and seen within *<* 15 days	(Number of COPD patients with radiological alert referred to Pulmonology from PC and seen within *<* 15 days/Total number of COPD patients with radiological alert seen in Pulmonology) × 100	>90%	Patient refusal of referral
Percentage of stable COPD patients referred from Pulmonology to Primary Care	(Number of stable COPD patients at levels I or II referred from Pulmonology to PC/Total number of stable COPD patients at levels I or II) × 100	>80%	Patient refusal of referral

AAT, alpha1-antitrypsin; COPD, chronic obstructive pulmonary disease; LAT, lateral; PA, posterior-anterior; PC, primary care.

## Data Availability

Not applicable.
